# Supplementary material for: Patterns of Intron Gain and Loss in Fungi
Source: PLoS Biol. 2004 Nov 30;2(12):e422. doi: 10.1371/journal.pbio.0020422 (PMC532390; doi:10.1371/journal.pbio.0020422)
Supplement: Table S1 — Also available at http://genes.mit.edu/NielsenEtAl/. (4.3 MB ZIP). [file pbio.0020422.st001.zip › NielsenEtAl/html/226.html]

AN2113.1.NCU02739.1.MG04821.1.FG00445.1


```
 CLUSTAL W (1.82) Multiple Sequence Alignments - Introns Inserted


Sequence 1: MG04821.1	478 aa
Sequence 2: FG00445.1	460 aa
Sequence 3: NCU02739.1	453 aa
Sequence 4: AN2113.1	377 aa
Alignment Length: 516 aa
Number Identitical Residues: 140 aa
Alignment Score (without introns) 8198


MG04821.1 	MPPRTSALTSSFSVSDSSLNEVICPLRNQD1GSSCRKRCIG0EKRYRSMQEHIRRAHPEH
NCU02739.1	MPPR-TSLTSSFSITDA-NNEVVCPLRNQD~GSSCRKRCIG0EKRYRSMQEHIRRAHPEH
FG00445.1 	MPPR-SALTSSFSITDS-NNEVVCPLRNQD~GSSCRKRCIG0EKRYRSMQEHIRRAHPEH
AN2113.1  	MPPR-STLTSSFSVSDT-NNEVVCPLKNND~GSNCRKRCLG0EKRYRSMQEHIRRAHPNH
          	**** ::******::*:  ***:***:*:* **.*****:* ****************:*

MG04821.1 	YISKLPATEESFLLMINTPPSERPQQSPATAP-----------RPPAIDHLKGSGYSHDR
NCU02739.1	YISKLPATEESFLLMIKTQPNERPQNQSTPGP------------SAHAGQAKG------L
FG00445.1 	YISKLPATEESFLLMINTPPSERRPLDQTSAPNAQGLSHSQSSLPPPIDHLQG---FHER
AN2113.1  	YIPKLPATEESFILMVTTPPEQRANLT-----------------PPQTARRRN-------
          	**.*********:**:.* *.:*                     ..   : :.       

MG04821.1 	Q~HGYHRDESSAPPTPRN-PEEYP---PVT-QMLPAASAAAALAQLHKHSMEPEWG----
NCU02739.1	S~HAYHRDGSSAPGTPRNGEEQYTG--------AALLPAATALAQLHNHKLEQGW-----
FG00445.1 	H~N-YHRDDSSNPGTPRL-IDEYTNGGPVSGPMLGTASAAAALAELHGVKSEREM-----
AN2113.1  	V1DRDLYAGSNSPATPRGIDEAHP----------AAATAAVALAQLHHNHLTPSWDTDVV
          	  .      *. * ***   : :.             .**.***:**        .:.  

MG04821.1 	-----------DSEP0GWHSDSEKGRLP-RSSIELPPIHLLGTDSAGDLFTMSGSSSRHR
NCU02739.1	-----------ESDN0DWHSDHEGKRRP-RSSIELPPIHLTNADVTSAPYS-GYDSGRPR
FG00445.1 	-----------DMDG0EYYSDMDVRRRP-RTSIELPPLNLPNHDITSDPYS-SAKANRQR
AN2113.1  	GAYIFSTDKMINETQ~EIHSDNDIDRAPMRSSIELPSLRDHFKRDSLPPFS----SPQPR
          	.:   ::..  :      :** :  * * *:*****.:.      :   ::    : : *

MG04821.1 	DMLPSILATSPPGRSSTLPPLQRPLGPNRPRKQSITKRGRESHHKKQRSRGAAADWLRRI
NCU02739.1	EILPSILSNSPPGRSSTLPPLHRTLGPTRTRRQSISKRG----HQGKRSKGATSEWLRRL
FG00445.1 	DFLPSILANSPPGRSSTLPPLQRPIGPNRPRKQSVTKRGRESHHKKKTSKGTATDWLRRI
AN2113.1  	SLLPSILNHSPPGRSSTLPPIQRTNKLPRPRKGSITGAR-----KAKHERSRSKEFGR--
          	.:*****  ***********::*.    *.*: *::        : : .:. : :: *  

MG04821.1 	QNDTGGLTPGDRLRPGDPFSKAKSAEP---TGDTGRRWEDLIDAA---ASATEEVDEDRT
NCU02739.1	QND----SQHELLRPGGSDRKASSAEP---SADFGKRWEDLIDAA---TSATEDIDEDRT
FG00445.1 	QNE-------ERYRPGN-DRKALSAEP---SADFGKRWEDLIDAADQAASAAGDIDEDRT
AN2113.1  	-------------RPSIGDRKALSAEPQTAAWAQGKRWEDLIEAA----TSATEVDDD--
          	             **.    ** ****.:::   *:******:**    ::: ::*:*  

MG04821.1 	P~VGPRSPYLIDPALTFQVPQSPISVHRASLPPFPQPPSFPSQNNYQASPLQQALTPPSY
NCU02739.1	P~VSSQGPAKFNPRLTSQIPRSPVSIHRSSLPPLQQHYNY---TSYQASPLQQALTPPSY
FG00445.1 	P0V----------------PQSPVSMHRSSLPPFSHQPQFQPAS-YQASPLQQALTPPSY
AN2113.1  	-~-----------------RQSEVGELIS--PDFTK---------FYLTYVVGAFTNHST
          	                    :* :.   :  * : :         :  : :  *:*  * 

MG04821.1 	IQEVPDPFPSVESAESGDNFHMESRGLSDSSPSYSSQNTQIYCAACQGVSLLKESYACTE
NCU02739.1	NPEATDAFPSVESGESGENFHIGSRGLSDSSPSYSSQNTQIYCAACREVSLLRESYACTE
FG00445.1 	SQDTIDPFPSVE---SGENFHMGTRGLSDSSPTYSAQNIQIYCAACQGFSLLKDSYACTE
AN2113.1  	NHS---------------EYRFDHIGTINQKP--------IIHAAC-----LPIPWATPT
          	  .               ::::   *  :..*        *  ***     *  .:* . 

MG04821.1 	CICGLCPACVEVLMSEQGARRKCPRCATIGGRYKPFQLDIR
NCU02739.1	CICGLCRACVDVLMLEQGARRKCPNCATIGGRFKPFQLDIR
FG00445.1 	CICGLCPTCVEVLMTEHGARRKCPRCATIGGRFKPFQLDIR
AN2113.1  	CIASPVPTHIICSVA-------------LAQIFDPAAL---
          	**..   : :   :              :.  :.*  *
```
